# MECP2e1 isoform mutation affects the form and function of neurons derived from Rett syndrome patient iPS cells

**DOI:** 10.1016/j.nbd.2015.01.001

**Published:** 2015-01-30

**Authors:** Ugljesa Djuric, Aaron Y. L. Cheung, Wenbo Zhang, Rebecca S. Mok, Wesley Lai, Alina Piekna, Jason A. Hendry, P. Joel Ross, Peter Pasceri, Dae-Sung Kim, Michael W. Salter, James Ellis

**Affiliations:** 1 Program in Developmental & Stem Cell Biology, The Hospital for Sick Children, Toronto, Ontario, M5G 0A4, Canada; 2 Department of Molecular Genetics, University of Toronto, Toronto, ON, M5S 1A8, Canada; 3 Program in Neurosciences and Mental Health, The Hospital for Sick Children, Toronto, ON, M5G 0A4, Canada; 4 Department of Physiology, University of Toronto, Toronto, ON, M5S 1A8, Canada; 5 University of Toronto Centre for the Study of Pain, University of Toronto, Toronto, ON, M5T 1P8, Canada

## Abstract

*MECP2* mutations cause the X-linked neurodevelopmental disorder Rett Syndrome (RTT) by consistently altering the protein encoded by the *MECP2e1* alternative transcript. While mutations that simultaneously affect both *MECP2e1* and *MECP2e2* isoforms have been widely studied, the consequence of *MECP2e1* deficiency on human neurons remains unknown. Here we report the first isoform-specific patient induced pluripotent stem cell (iPSC) model of RTT. RTTe1 patient iPS cell-derived neurons retain an inactive X-chromosome and express only the mutant allele. Single-cell mRNA analysis demonstrated they have a molecular signature of cortical neurons. Mutant neurons exhibited a decrease in soma size, reduced dendritic complexity and decreased cell capacitance, consistent with impaired neuronal maturation. The soma size phenotype was rescued cell-autonomously by *MECP2e1* transduction in a level-dependent manner but not by *MECP2e2* gene transfer. Importantly, *MECP2e1* mutant neurons showed dysfunction in action potential generation, voltage-gated Na^+^ currents, and miniature excitatory synaptic current frequency and amplitude. We conclude that *MECP2e1* mutation affects soma size, information encoding properties and synaptic connectivity in human neurons that are defective in RTT.

## INTRODUCTION

Rett Syndrome (RTT) is a neurodevelopmental disorder [OMIM312750] characterized by repetitive hand motions and loss of acquired language (Chahrour and Zoghbi, 2007). Heterozygous loss-of-function mutation in the X-linked gene encoding Methyl-CpG Binding Protein 2 (*MECP2*) is the prime cause of RTT in girls (Amir et al., 1999). This gene is alternatively spliced into *MECP2e1* and *MECP2e2* isoforms that encode distinct proteins differing at the N-termini due to exclusion or inclusion of exon 2 respectively (Kriaucionis and Bird, 2004; Mnatzakanian et al., 2004). Mutations that affect both isoforms have been widely studied, and the role of MECP2 in binding methylated and hydroxy-methylated cytosine genome-wide (Mellen et al., 2012; Skene et al., 2010) to recruit chromatin-remodelling proteins that modulate global transcription is now well established (Chahrour et al., 2008; Lyst et al., 2013). TALEN-mediated mutagenesis of the *MECP2* locus demonstrated that MECP2 ablation results in global decreases in gene transcription and translation in a human ES cell-based model of RTT (Li et al., 2013). These defects were manifest in abnormal neuronal morphology and function, including impaired mitochondrial function.

The majority of RTT patient mutations affect both isoforms but identification of individuals with a *MECP2e1*-specific mutation that does not alter *MECP2e2* indicates that MECP2e1 isoform dysfunction is sufficient to cause RTT (Mnatzakanian et al., 2004). A recent report of a *Mecp2e1*-specific mouse model of RTT further suggests that this MECP2 isoform is responsible for RTT-related behavioural abnormalities (Yasui et al., 2014), but the effect of MECP2e1 deficiency on human neurons has not be evaluated. In contrast, no *MECP2e2*-specific mutations have been described in RTT patients, and *Mecp2e2*-mutant mice lack neurological phenotypes and instead exhibit placental defects (Itoh et al., 2012). However, expression of either *Mecp2e1* or *Mecp2e2* can improve a subset of RTT-related behavioural phenotypes in *Mecp2*-null mice (Kerr et al., 2012). These findings suggest that endogenous *MECP2e1* is essential for normal brain function, but *Mecp2e2* can ameliorate certain disease features in mouse models of RTT.

We, and others, reported the generation of human and mouse induced pluripotent stem cells (hiPSCs and miPSCs, respectively) from RTT patients and mouse models that carry pathogenic mutations in both *MECP2* isoforms (Ananiev et al., 2011; Cheung et al., 2011b; Kim et al., 2011). RTT iPSC-derived neurons exhibit maturation and electrophysiological defects reminiscent of those seen in RTT patients and mouse models (Cheung et al., 2012; Farra et al., 2012) and are amenable to rescue by introduction of exogenous MECP2 or drugs such as IGF1 (Li et al., 2013; Marchetto et al., 2010). Generally, female RTT-hiPSCs retain an inactive X-chromosome (Xi) (Pomp et al., 2011; Tchieu et al., 2010) and express either the wild-type (WT) or mutant *MECP2* allele and this expression pattern is conserved upon differentiation into neurons (Cheung et al., 2012). Here, we generated hiPSC-derived neurons that express mutant *MECP2e1*. Using this system, we find that *MECP2e1* mutation affects the soma size and electrophysiological properties of human neurons.

## Materials and Methods

### Cell Culture

RTTe1-fibroblasts were obtained from Dr. Patrick Macleod at the Victoria General Hospital, Victoria, BC, Canada, and cultured under the approval of the SickKids Research Ethics Board and Canadian Institutes of Health Research Stem Cell Oversight Committee. Fibroblasts were maintained in fibroblast medium: Dulbecco’s Modified Eagle Medium (DMEM) containing 10% Fetal Bovine Serum, and 100X Penicillin and Streptomycin (all from Invitrogen). RTTe1-hiPSCs were generated from fibroblasts and maintained in hiPSC medium as previously described (Hotta et al., 2009).

### Androgen Receptor Assay

To identify the methylated Xi, 200 ng of DNA was digested overnight at 37°C with methylation-sensitive enzymes *Hpa*II and *Hha*I (Invitrogen). To discriminate between the two parental X-chromosomes, 20 ng of digested and undigested DNA was amplified with primers spanning the heterozygous polymorphic trinucleotide repeat in the first exon of the *AR* gene for 32 cycles. The 5′ end of the forward primer was labelled with FAM fluorescein (Invitrogen). PCR products were separated on an ABI3100 Genetic Analyzer with 500 LIZ size standard and analysed by Peak Scanner software (all from Applied Biosystems). XCI ratio (Table S1) was calculated as previously described (Cheung et al., 2011b).

### *In vitro* and *in vivo* differentiation

For *in vitro* differentiation, hiPSCs were detached and grown in suspension in hiPSC medium (Hotta et al., 2009) without FGF2 for eight days to form embryoid bodies. Embryoid bodies were adhered and allowed to further differentiate for eight days. Differentiated derivatives were analysed via immunocytochemistry with appropriate antibodies (Table S4). For *in vivo* differentiation, one 10 cm dish of hiPSCs were detached and suspended in a mixture of KNOCKOUT DMEM (Invitrogen), Matrigel (BD Biosciences), Collagen (STEMCELL Technologies) (ratio 2:1:2), and 10 μM ROCK Inhibitor (Sigma) and injected intramuscularly into immunodeficient mice. Fixed tumours were embedded in paraffin, sectioned, and stained with hematoxylin and eosin for pathological analysis. All procedures using animals were approved by the SickKids Animal Care Committee under the auspices of The Canadian Council on Animal Care, and conducted with the approval of the Canadian Institutes of Health Research Stem Cell Oversight Committee.

### Immunocytochemistry

Cells were fixed with 4% formaldehyde (EMD Biosciences) for 10 min at room temperature (RT), permeabilized with 0.1% Noniodet P-40 (Sigma). Blocking was performed for 3 hr at RT, primary antibodies diluted in block solution and incubated overnight at 4° C (See Table S4 for antibodies used). Images were captured using a Leica DMI4000B microscope equipped with Leica DFC340FX camera and Leica Application Suite software for hiPSCs or Zeiss Axiovert 200M microscope equipped with a Hamamatsu C9100-13 EMCCD camera and Improvision Volocity software for neurons. Soma size of neurons was scored using Improvision Volocity software on 40X images blinded to the observer.

### Neuronal Differentiation

The Brennand protocol with slight modifications (Brennand et al., 2011; Chambers et al., 2009) was used for neuronal differentiation of the RTTe1 #27 line (Table S2). The Kim protocol with slight modifications (Kim et al., 2012) or the Brennand protocol with the addition of DAPT in the medium was used for the remaining lines (Table S2). Analysis was compared to WT-neurons generated with the same protocol. Soma-size measurements were performed as described (Cheung et al., 2011b). See supplemental material for more details.

### Generation and transduction of lentivirus

*MECP2* isoform-specific lentiviruses were previously reported (Rastegar et al., 2009). The generation and transduction of lentivirus were performed as previously described (Hotta et al., 2009). In brief, plasmids containing cDNA of interest were transfected into 293T cells using Lipofectamine 2000 (Invitrogen). The supernatant containing virus was collected two days post-transfection and concentrated by ultracentrifugation. 293T cells were maintained in Dulbecco’s Modified Eagle Medium (DMEM) containing 10% Fetal Bovine Serum, and 100X Penicillin and Streptomycin, MEM NEAA (all from Invitrogen). Titration of lentivirus was performed as previously described (Hotta et al., 2009). In brief, 293T cells were transduced with EF1α-EGFP lentivirus. Titer (infectious units, IU) of lentivirus was calculated with the following formula: Viral titer (IU ml^−1^) = [Infected cell number] x [EGFP^+^%/100]/[Amount of virus used (ml)]. Transduction of NPCs was performed in the presence of 0.6 μg ml^−1^ Hexadimethrine bromide (Sigma) for six hours with a multiplicity of infection (MOI) of one calculated for 293T cells.

### RNA Isolation, RT-PCR and cDNA Sequencing

RNA was isolated using Trizol extraction method (Invitrogen) and SSII RT (Invitrogen) was used for the reverse transcription following manufacturer’s instructions. Primers for the Real-Time PCR assays were designed using Primer3 online primer design software and qPCR was carried out using SYBR green (Applied Biosystems) on an ABI 7900HT PCR System (Applied Biosystems). To sequence RTTe1-hiPSC cDNA, RT-PCR was performed using V1 primers (Table S3). The *MECP2e1* amplicon (382 bp or 371 bp for WT or mutant allele, respectively) was gel purified from the *MECP2e2* amplicon (506 bp) using QIAquick PCR Purification Kit following manufacturer’s instructions and sequenced using the V1-f for amplification.

### Bisulfite Sequencing

Bisulfite conversion was performed as previously described (Fussner et al., 2011). Briefly 1 μg of DNA was subjected to conversion using the DNA Methylation Gold Kit (Zymo Research). 50 ng of converted DNA was subjected to PCR with appropriate primers (Table S3).

### Single-cell Fluidigm Array

Neurons were subjected to single cell sorting and Fluidigm analysis as described (Pasca et al., 2011). Single cell sorting was performed by The Flow Cytometry Facility at The Hospital for Sick Children (Toronto, Ontario, Canada) using MoFlo BRU cell sorter (Beckman Coulter). Cells were stained by propidium iodide and live cells were sorted into CellsDirect amplification master mix (Invitrogen) into 96-well plates. RT reaction and 15 rounds of cDNA amplification were performed using primers to genes of interest (Table S3). Fluidigm Biomark qPCR assays were performed by Janet Rossant lab at The Hospital for Sick Children (Toronto, Ontario, Canada). Data analyses and generation of figures were performed using R-Statistical analysis software (http://cran.r-project.org). For neuronal identity quantifications, cells were deemed neurons if expressing *NCAM* or *MAP2* with: *FOXP1* and *ETV1* double positive (lower layer); *FOXP1-/ETV1-/CUX1+/SATB2+/CTIP2+/REELIN+* (upper layer); *CAMK2+, VGLUT1+, VGLUT2+* or *VGLUT3+* (glutamatergic); *GAD67+* or *VGAT+* (GABAergic) (Pasca et al., 2011).

### Western Blot Analysis

Nuclear proteins were collected using nuclear extraction protocol, samples were aliquoted and snap frozen in liquid nitrogen for storage until western blots were performed. 5 ug of total protein was loaded for western blots, transferred to nitrocellulose membranes overnight at 4 degrees. Membranes were blocked in 5% Milk PBS-T and incubated overnight in primary antibodies (see Table S4), washed 5 times in PBS-T and incubated in appropriated HRP-conjugated secondary antibodies (Invitrogen) for 1 hour at room temperature. Densitometry measurements and normalization (to Histone H3 signal) was performed using ImageJ software.

### Karyotyping

Standard G-banding chromosome analysis with a 400–500 banding resolution was performed at The Centre of Applied Genomics (TCAG [The Hospital for Sick Children, Toronto, Canada]).

### Electrophysiology

Electrophysiology was performed as described (Farra et al., 2012). See supplemental information for detailed protocol.

## RESULTS

### Isolation of Mutant RTTe1-hiPSCs Through XCI

**Figure 1:**
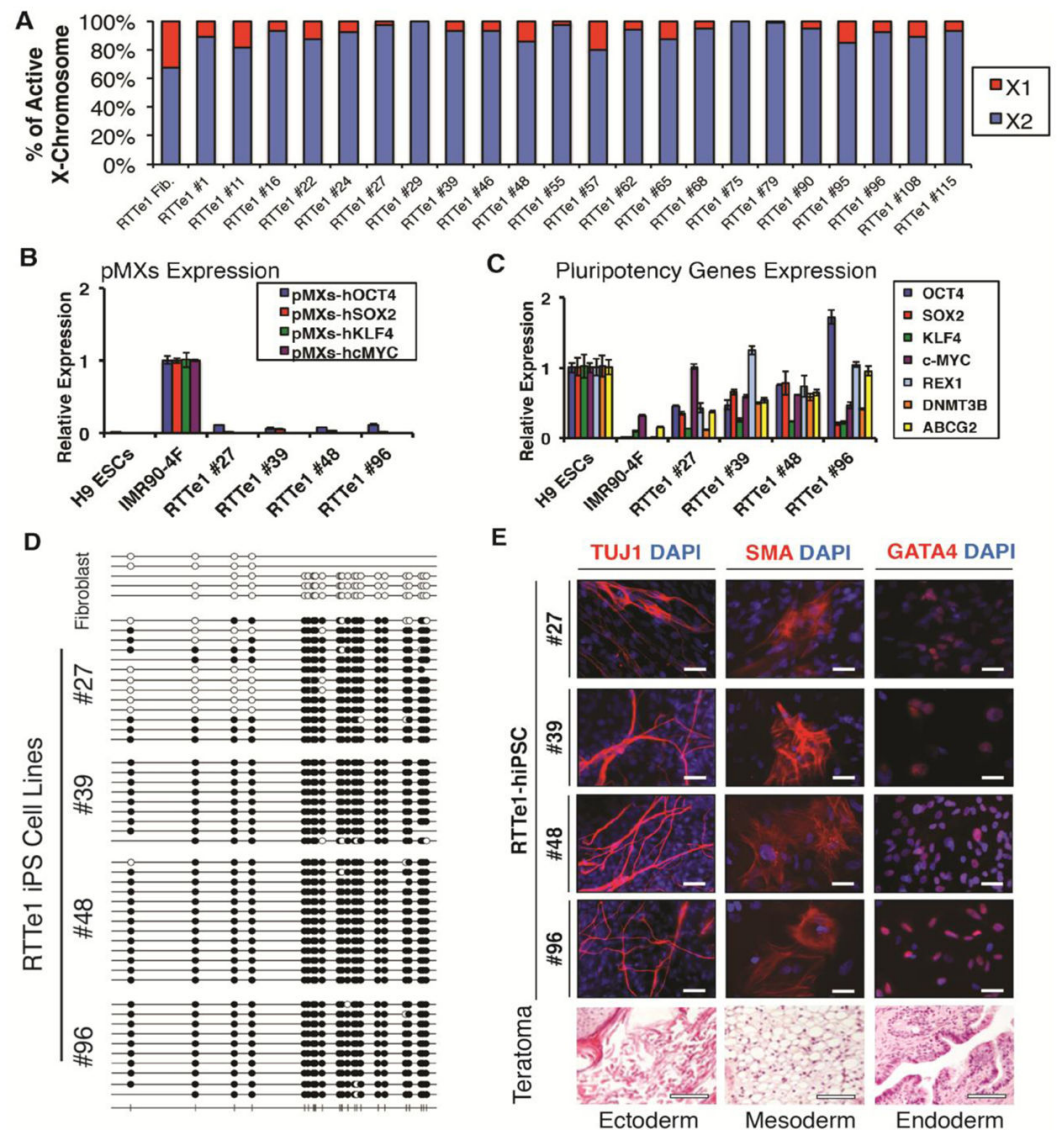
Generation and pluripotency characterization of RTTe1-hiPSCs (**A**) Androgen receptor (AR) methylation screen of RTTe1-hiPSCs demonstrates skewed XCI ratios in all examined lines. Bar graph depicts XCI ratio of two X chromosomes (X1 and X2). Fib., fibroblasts. (**B**) qRT-PCR analyses of pMXs reprogramming retroviral transgenes. (**C**) qRT-PCR of endogenous pluripotency genes in RTTe1-hiPSC lines. Data are expressed as mean ± SEM. (**D)** Bisulfite sequencing of retroviral pMXs-LTR reprogramming vectors in RTTe1-hiPSC lines. Open and closed CpG sites indicate unmethylated and methylated CpG sites, respectively. **(E)** RTTe1-hiPSCs differentiate into the three germ layers *in vitro* and *in vivo* (representative images of RTTe1-hiPSC #39 teratomas). Scale bars, 50 μm (immunocytochemistry) and 100 μm (histology).

**Figure 2:**
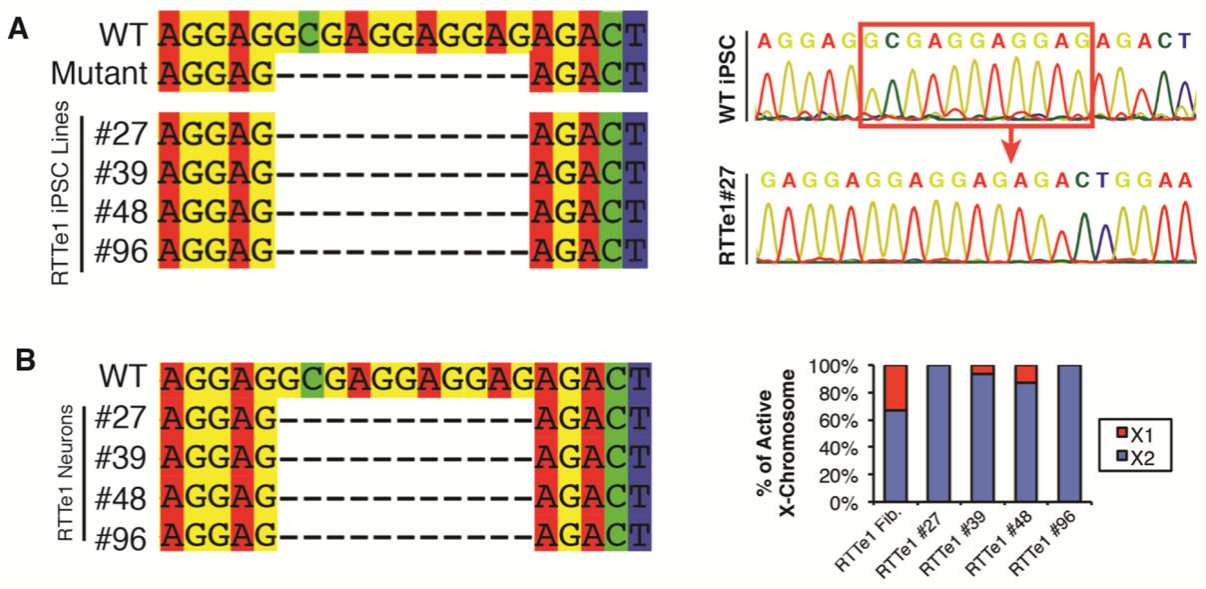
RTTe1-hiPSCs and -neurons retain an Xi and exclusively express the mutant *MECP2e1* allele (**A**) cDNA sequencing of *MECP2e1* transcripts in selected RTTe1-hiPSCs reveals that the expressing X-chromosome contains the 11 bp RTTe1 deletion (left panel), with representative chromatogram (right panel). (**B**) Differentiated RTTe1-neurons maintain expression of the mutant *MECP2* allele (left panel) and share the same inactive X chromosome revealed by the AR assay (right panel).

Fibroblasts acquired from an RTT patient with an 11 base-pair (bp) deletion in exon 1 of *MECP2* (RTTe1) were used to generate RTTe1-hiPSCs via retroviral transduction of human *OCT4*, *SOX2*, *KLF4*, and *c-MYC*. This exon 1 specific NM_001110792.1:c.47_57del; p.(Gly16Glufs*22) mutation causes a frameshift resulting in severely truncated *MECP2e1* but intact *MECP2e2* (Mnatzakanian et al., 2004). We used the Androgen Receptor (AR) assay to determine the X-inactivation status in RTTe1-hiPSCs, revealing that all of the 22 RTTe1-hiPSC lines exhibited XCI skewing (80:20- ~ 100:0) towards the same parental X-chromosome (Fig. 1A, Table S1). We selected four RTTe1-hiPSC lines for detailed pluripotency characterization (Table S2). All four hiPSC lines silenced and methylated the reprogramming retroviral transgenes, activated the pluripotency-related genes (Figs. 1B–D) and had normal karyotypes (Fig. S1). Embryoid body-mediated spontaneous *in vitro* and teratoma-based *in vivo* differentiation assays demonstrate that RTTe1-hiPSC lines give rise to cells of all three germ layers (Fig. 1E). Collectively these data demonstrate that the generated hiPSC lines are of high quality appropriate for RTT disease modelling. To determine the identity of the active X-chromosome, we performed *MECP2* cDNA sequencing with primers spanning the 11 bp deletion revealing that the RTTe1-hiPSCs solely expressed the mutant allele (Fig. 2A). To ensure that neither a change in the Xi nor Xi erosion (Mekhoubad et al., 2012) occurred during differentiation, we confirmed persistent expression of the mutant *MECP2e1-*containing X-chromosome using cDNA sequencing and the AR assay on hiPSC-derived neurons (Fig. 2B) (see below for neuronal characterization).

### Directed Differentiation Into Cortical Neurons Expressing Mutant *MECP2e1*

**Figure 3:**
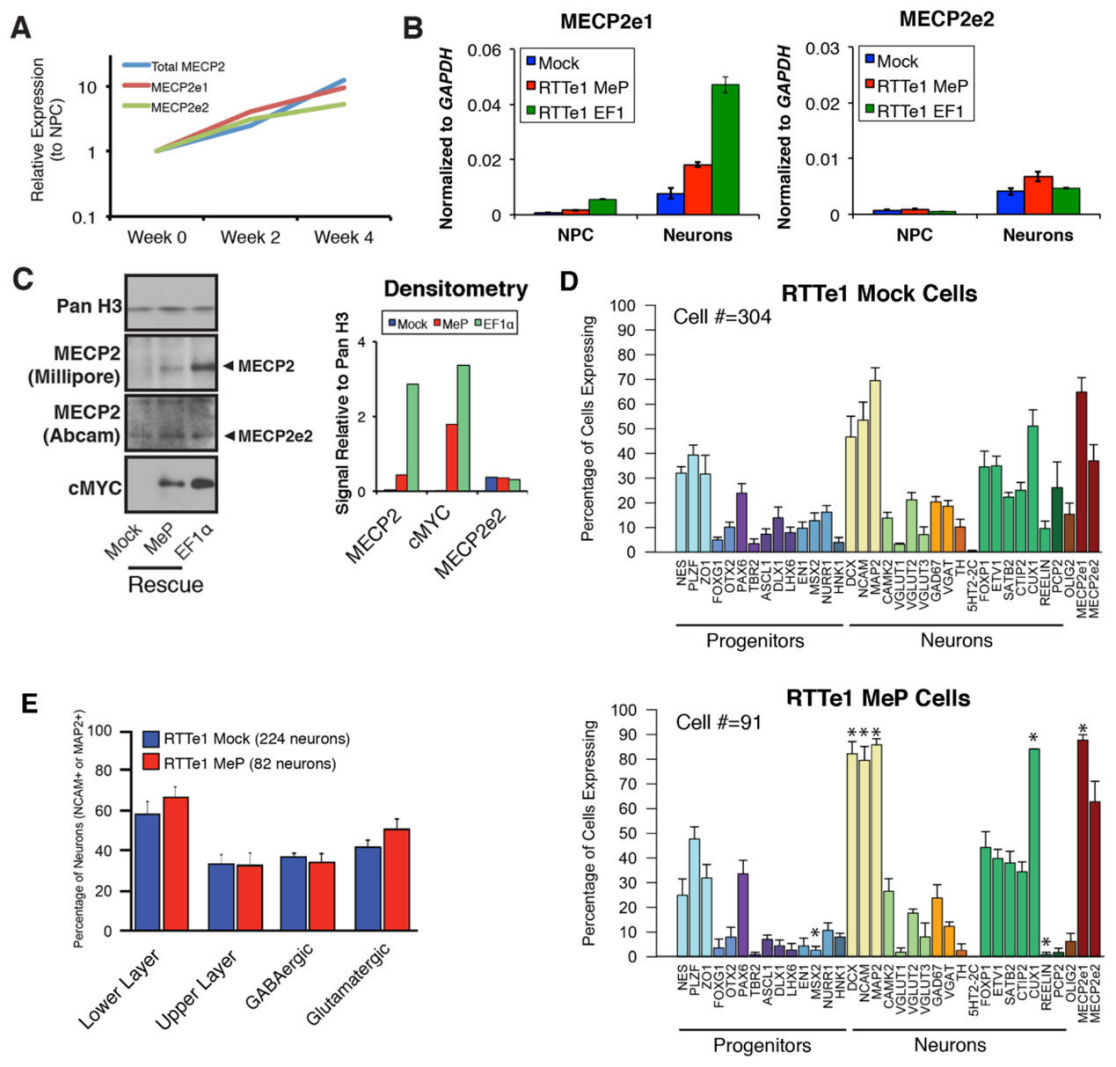
RTTe1 neurons maintain normal balance of neuronal identities (**A**) Transcription of both *MECP2* isoforms increases over the course of neuronal differentiation. (**B**) MeP-*MECP2e1* and EF1α-*MECP2e1* lentiviral constructs are expressed in mature RTTe1#27 neurons. Data are expressed as mean ± SEM. (**C**) Western blot analysis of transduced RTTe1#27 cells shows that MeP-*MECP2e1* rescue results in a moderate increase of total MECP2 protein, and detectable levels of the MYC tagged MECP2e1 with unchanged levels of MECP2e2 protein. Transduction with EF1α-*MECP2e1* construct leads to overexpression of total MECP2 protein and higher levels of MYC tagged MECP2e1. Densitometry bar graph is shown with normalization to loading control, histone H3. (**D**) Bar graphs show comparable percentage of cells expressing majority of neuronal markers as determined by Fluidigm arrays in RTTe1-mock (cumulative data from all 4 RTTe1 lines) and MeP rescued RTTe1#27 cells. Data are expressed as mean ± SEM; * p-value<0.05. (**E**) Similar percentages of cortical layer and neurotransmitter neurons are produced from both RTTe1-mock and MeP rescue cells. Data are expressed as mean ± SEM; * p-value<0.05.

To generate neurons *in vitro*, we found that the Brennand protocol efficiently differentiated the RTTe1 #27 line into neuronal progenitor cells (NPCs) and neurons while the remaining three RTTe1-hiPSC lines required optimization using previously described protocol variations (Table S2) (Brennand et al., 2011; Kim et al., 2012). Cumulative results using neurons derived from all four RTTe1-hiPSC lines are shown for subsequent experiments, and the RTTe1 #27 line was utilized for all genetic rescue studies. We observed a 10-fold increase in *MECP2* mRNA upon transition of RTTe1-NPCs into neurons (Fig. 3A). Since we were unable to establish RTTe1-hiPSCs expressing the wild-type allele, we compared RTTe1-neurons to those transduced by a lentiviral vector expressing *MECP2e1*-MYC under the control of the *Mecp2* promoter (MeP) or the ubiquitously expressed EF1α promoter (Rastegar et al., 2009). Vector transduction resulted in specific increase of *MECP2e1* mRNA in differentiated MeP neurons (Fig. 3B). Total MECP2 protein moderately increased in MeP transduced cells, and was higher in the EF1α transduced cells while MECP2e2 protein levels remained unaffected (Fig. 3C). The relative levels of exogenous MECP2 are most easily quantified by densitometry using the MYC antibody which has a cleaner signal than MECP2, indicating that the EF1α vector expresses roughly twice as much MECP2e1-MYC protein as MeP in neurons similar to the qRT-PCR data shown in Fig 3B. These results demonstrate that RTTe1 cells continue to express *MECP2e2* while the vector transduced cells express different levels of exogenous MECP2e1 protein.

To investigate neuronal identity of the differentiated RTTe1-hiPSC lines, we characterized single cell mRNA profiles using Fluidigm arrays (Pasca et al., 2011). More RTTe1-MeP cells were expressing the mature neuronal markers *DCX*, *NCAM* and *MAP2* compared to RTTe1 mock infected cells but, importantly, both RTTe1 mock and RTTe1-MeP cells were of comparable neuronal types (Fig. 3D). These included cells with a dorsal forebrain identity indicated by *PAX6* expression, a roughly 60:30% distribution of lower and upper cortical layer neurons, respectively; and an equal (~35:45%) mixture of glutamatergic and GABAergic neurons (Fig. 3E), based on concurrent expression of region- or neurotransmitter-specific genes from single-cell Fluidigm arrays (see Materials and Methods). To determine the consequences on neurons that lack both isoforms, we compared neurons derived from previously generated *MECP2* null iPS cells (Δ3-4#20) to their isogenic (Δ3-4#37) *MECP2* WT-neurons (Cheung et al., 2011b). The Fluidigm array results showed that complete absence of *MECP2* also did not dramatically alter the type of neurons generated (Figs. S2A and S2B). Collectively, these results indicate that lack of *MECP2e1* has minimal effects on neuronal differentiation fate *in vitro* and that cortical neurons relevant to RTT phenotypes were generated.

### MECP2e1 is a Level-Dependent Cell Autonomous Regulator of Soma Size

**Figure 4:**
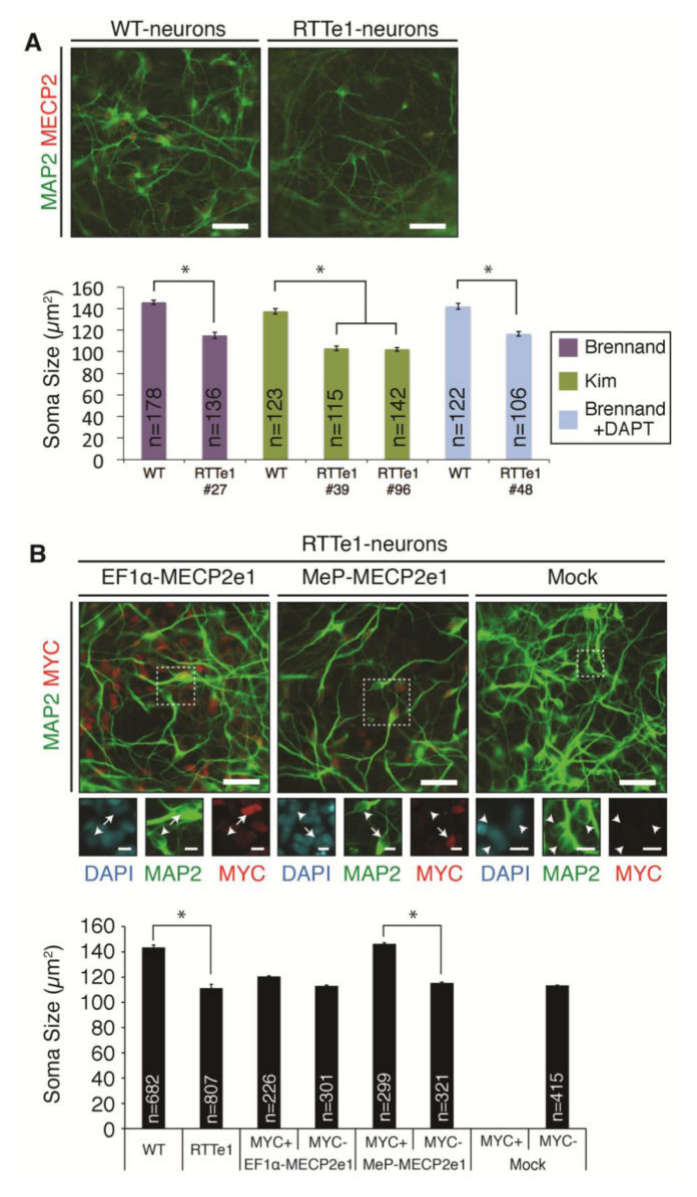
RTTe1-neurons exhibit a soma size defect that can be rescued by *MECP2e1* in a cell-autonomous manner (**A**) Immunocytochemistry for MAP2 and MECP2 in WT Δ3-4#37 and RTTe1#27 neurons. Bar graph represents soma size analysis of neurons derived from individual RTTe1 hiPS cell lines compared to WT neurons derived by three differentiation protocols utilized in the study. (**B**) Immunocytochemistry for MAP2 and MYC in RTTe1#27-neurons with or without *MECP2e1*-vectors. Bar graph shows cumulative soma size analysis of all four RTTe1-neurons, and RTTe1#27 neurons with MECP2e1-vectors compared to WT-neurons. Total number of measured neurons for each analyzed genotype is indicated within the appropriate bar. Data are expressed as mean ± SEM (*P < 0.001; Student’s *t*-test; *n* = at least 3 independent differentiations). Scale bars, 44 μm for large image, 10 μm for inset. Arrows, MYC-positive neurons. Arrowheads, MYC-negative neurons.

RTT is thought to be a neurodevelopmental disorder with defects in neuronal maturation and/or maintenance (Kishi and Macklis, 2004; Nguyen et al., 2012). To determine whether the loss of *MECP2e1* alone results in a neuronal phenotype, we measured soma size in RTTe1-neurons. All 4 RTTe1-hiPSCs lines were differentiated into MAP2-positive neurons, with negligible MECP2, consistent with continued low-level expression of *MECP2e2* and showed significant decrease in soma size compared to WT-neurons derived from Δ3-4#37 (Fig. 4A). Cumulative data of RTTe1-neurons derived from all 4 lines also showed a significant decrease in soma size compared to WT-neurons derived from Δ3-4#37 (Fig. 4B, bars 1 and 2). In addition, sparse transfection with an *EF1α-EGFP* reporter was used to label single neurons and confirmed that dendrite length, tip number and complexity is reduced in RTTe1-neurons relative to WT-neurons (Fig. S3). These RTT-associated morphological phenotypes suggest that MECP2e1 is important for neuronal maturation.

To test whether the easily measured soma size defect is due to *MECP2e1* mutation, RTTe1#27-NPCs were infected with *MECP2e1* lentiviral vectors prior to the final neuronal differentiation step. Transduced NPCs expressed the ubiquitous EF1α-vector while the MeP construct was only activated upon neuronal differentiation (Fig. S4A and S4B). These results demonstrate temporal regulation of the MeP promoter during maturation of human neurons *in vitro*. Co-staining with MAP2 and MYC revealed that roughly half of the RTTe1-neurons expressed lentiviral transgenes (Fig. 4B). Therefore, soma size was scored in MYC-positive relative to MYC–negative neurons. No statistically significant differences were observed in the EF1α-transduced neurons (Fig. 4B, bars 3 and 4). These neurons express twice the level of MYC tagged MECP2e1 protein (Fig 3C), which may be incompatible with rescue of neuronal morphology phenotypes similar to the finding that *MECP2* duplication causes neurological phenotypes. Only the *MeP-*transduced MYC positive neurons showed a statistically significant soma size increase in comparison to the adjacent MYC negative neurons (bars 5 and 6). Mock infected cells were all MYC-negative with small soma size (bars 7 and 8). These results reveal that soma size is rescued by *MeP-MECP2e1* in a cell autonomous manner.

**Figure 5:**
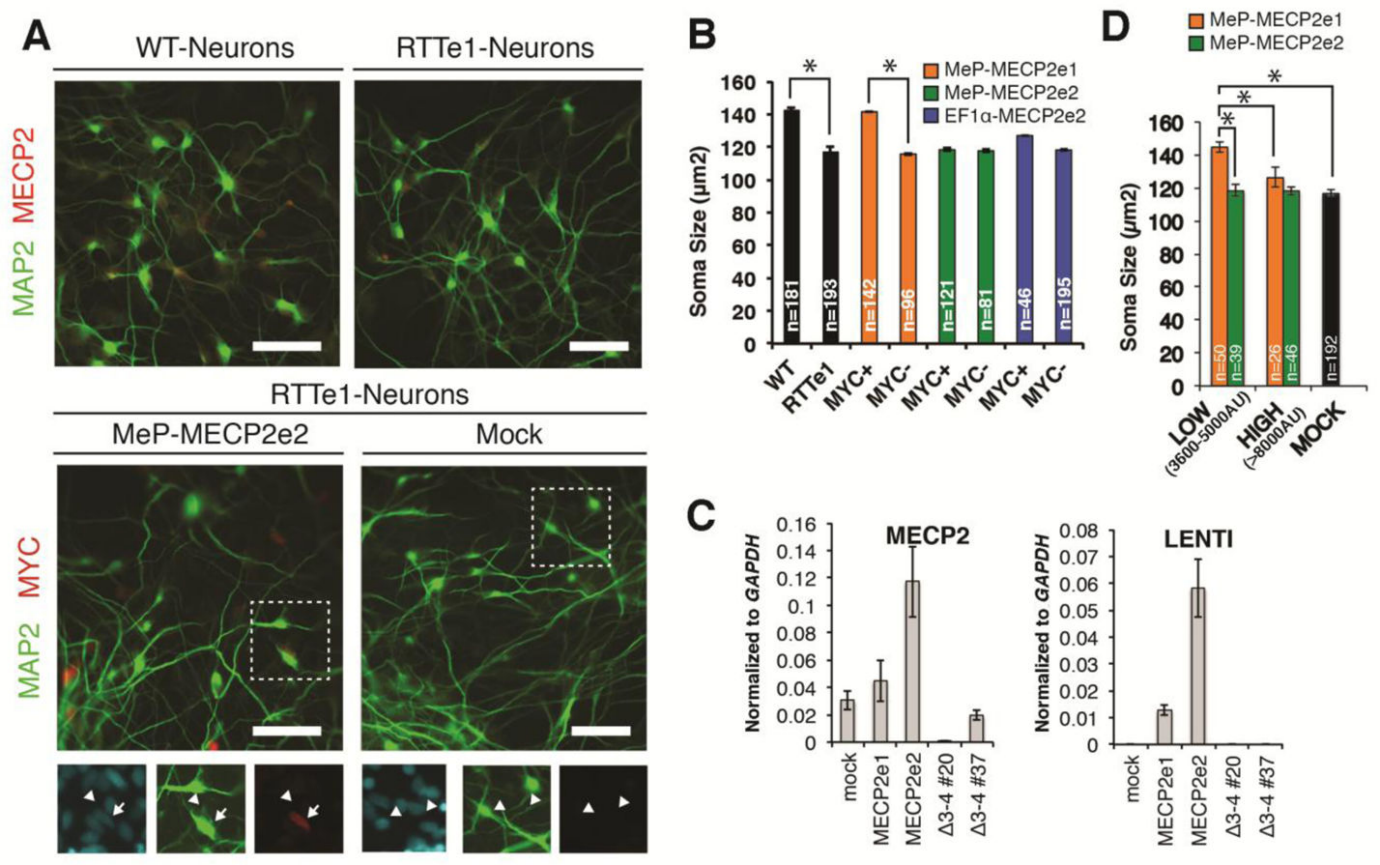
Soma-size rescue is MECP2e1 level-dependent (**A**) Immunocytochemistry for MAP2 and MECP2 or MAP2 and MYC in RTTe1-neurons with or without *MECP2e2*-vectors and WT-neurons. Scale bars, 44 μm for large image. Arrows, MYC-positive neurons. Arrowheads, MYC-negative neurons. (**B**) Soma size analysis of RTTe1-neurons with or without MECP2e1 (control) or MECP2e2 vectors compared to WT-neurons. Number of measured neurons for each analyzed genotype is indicated within the appropriate bar. (**C**) qRT-PCR of total *MECP2* and the lenti-derived *MECP2* transcripts in cells transduced with either MECP2e1 or MECP2e2 vectors. (**D**) Soma size analysis in cells expressing low or high levels of MECP2, based on immunostaining intensities of the MYC signal. Data are expressed as mean ± SEM (*P < 0.001; Student’s *t*-test; *n* = 3 independent differentiations).

To assess whether the soma size phenotype is due to the specific absence of MECP2e1, we performed additional transductions of RTTe1#27-NPCs with *MECP2e2* vectors. MECP2e2 was poorly expressed by the MeP promoter in neurons, likely due to its shorter protein half-life (Yasui et al., 2014). Therefore, higher *MeP-MECP2e2* multiplicity of infection (MOI) was used to obtain MAP2 positive neurons with detectable MYC signals (Fig. 5A). As expected, the control MYC-positive *MeP-MECP2e1* transduced neurons rescued the soma size equivalent to WT-neurons and the *EF1α-MECP2e2* transduced neurons showed no statistically significant differences in soma size (Fig. 5B). Strikingly, the *MeP-MECP2e2* vector was unable to rescue (Fig 5B). We observed that the mRNA levels in *MeP-MECP2e2* transduced cells was greater than with *MeP-MECP2e1* (Fig. 5C) but the MYC intensity in MeP-*MECP2e2* transduced neurons was not (Fig 5B). Because soma size can only be measured in single cells, the most accurate comparison of the two rescue vectors was to identify single neurons expressing similar protein levels by binning intracellular immunofluorescence intensity levels of the MYC signal. MYC signal intensity covered the same range (3,600 – 30,000 arbitrary units) in both the *MeP-MECP2e1* and *MeP-MECP2e2* transduced neurons. We found that low expressing *MeP-MECP2e1* RTTe1 neurons (3,600 – 5,000 arbitrary units) have a soma size rescue that is statistically significantly different from the high *MECP2e1* expressing and mock infected cells, confirming that *MECP2e1* rescue is level-dependent (Fig. 5D). On the other hand, neither high (greater than 8,000 arbitrary units) nor the similarly low *MeP-MECP2e2* expressing RTTe1 cells had a soma size rescue. Since both isoforms contained the same C terminal MYC tag and were expressed from the same vector, the rescue is not due to tag or vector effects. We conclude that MECP2e2 is unable to efficiently rescue soma size in our system when comparing single cells expressing similar levels of the MECP2 isoform transgenes. These data indicate that MECP2e1 is a cell autonomous regulator of soma size.

### MECP2e1 Controls Action Potentials and Excitatory Synaptic Responses

**Figure 6:**
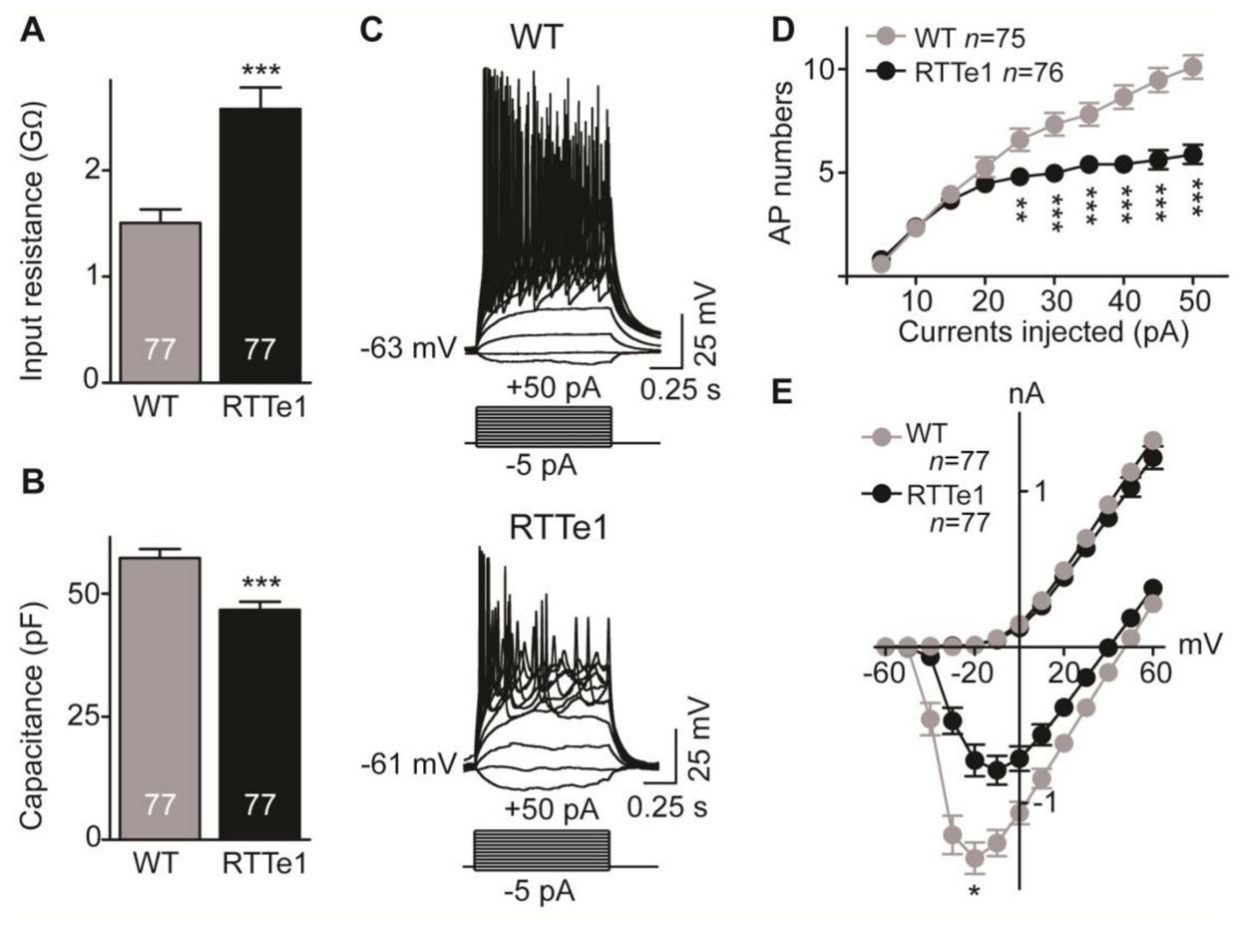
RTTe1-neurons exhibit alterations in intrinsic membrane properties (**A**) Histogram shows average input resistance in WT- and RTTe1-neurons. (**B**) Bar graph showing average cell capacitance in WT-neurons compared with RTTe1-neurons. (**C**) Representative traces show evoked action potentials triggered by injecting a series of current steps from −5 pA to +50 pA in WT- (**Upper**) and RTTe1- (**Bottom**) neurons. (**D**) A plot showing the numbers of action potentials evoked by injecting a series of current steps from +5 pA to +50 pA in WT- and RTTe1-neurons. (**E**) A plot depicting current-voltage relationships between WT- and RTTe1-neurons. Peak average inward currents (at −20 mV) were compared between WT- and RTTe1-neurons. *P<0.05, **P<0.01, ***P<0.001.

We next determined whether glutamatergic RTTe1-neurons exhibit any electrophysiological defects. Whole-cell patch-clamp recordings were performed on over 220 hiPSC-derived neurons from two WT (characterization of SK0019_002#7 hiPSCs is in Fig. S5) and four RTTe1 lines. Electrophysiological studies demonstrated RTTe1-neurons compared with WT-neurons had higher input resistance (Fig. 6A), and lower cell capacitance (Fig. 6B) dependent on the membrane area of the cells (Golowasch et al., 2009; Limon et al., 2005), corresponding to the decrease in soma size in RTTe1-neurons. There is no significant difference in the resting membrane potentials between WT- and RTTe1-neurons (Fig. S6A). These hiPSCs-derived cells had functional neuronal properties and generated tetrodotoxin-sensitive Na^+^ channel-mediated spontaneous and/or evoked action potentials (Fig. 6C, Fig. S6C and S6D). However, evoked action potentials in RTTe1-neurons exhibited smaller amplitude, longer time course (increased rise time, half-duration, and decay time), and fewer numbers as a series of current steps were injected (Fig. 6D, Fig. S6E–I). The defects in generating action potentials in RTTe1-neurons could be attributed to a decrease in voltage-gated Na^+^ currents. Indeed, significant decreases in amplitude and density of voltage-gated Na^+^ currents were observed in RTTe1-neurons (Fig. 6E, Fig. S6B). There was no significant difference in voltage-gated K^+^ currents between WT- and RTTe1-neurons. These findings indicate that RTTe1-neurons have deficiencies in intrinsic membrane properties similar to neurons with *MECP2* null mutation (Calfa et al., 2011; Farra et al., 2012; Zhang et al., 2010).

**Figure 7:**
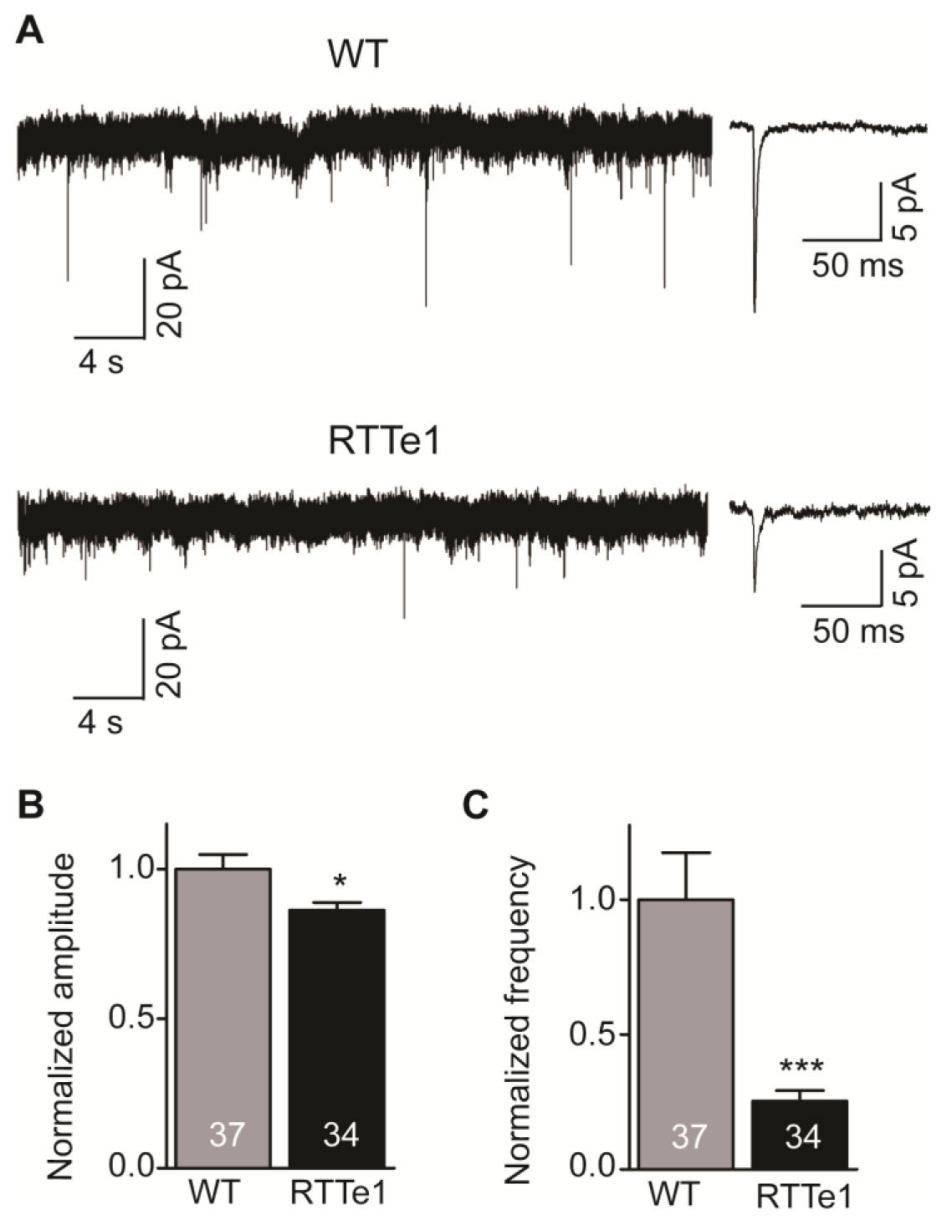
RTTe1-neurons exhibit decreased mEPSC frequency and amplitude (**A**) Representative traces showing mEPSCs in WT- (**Upper**) and RTTe1- (**Bottom**) neurons, and the inset showing averaged mEPSCs in the WT- (**Upper)** and RTTe1- (**Bottom**) neurons, respectively. (**B**) Bar graph shows average mEPSC amplitude in WT-neurons compared with RTTe1-neurons. (**C**) Histogram showing average mEPSC frequency in WT-neurons compared with RTTe1-neurons. *P<0.05, ***P<0.001.

The other evidence demonstrating the functional neuronal properties of these hiPSC-derived cells is that WT- and RTTe1-neurons displayed spontaneous synaptic activity (Fig. 7A and Fig. S7). Studies from us (Farra et al., 2012) and other investigators (Chao et al., 2007; Dani et al., 2005; Marchetto et al., 2010; Nelson et al., 2006) have shown that neurons with *MECP2* null mutation have synaptic defects. Thus, we recorded miniature excitatory postsynaptic currents (mEPSCs) to examine whether RTTe1-neurons have any defects in synaptic function. Estimates of mEPSCs uncovered that RTTe1-neurons displayed dysfunctional synaptic activities with significant decreases in both amplitude (Fig. 7B) and frequency (Fig. 7C) of mEPSCs. Collectively, these findings demonstrate that MECP2e1 controls glutamatergic neurophysiology and its loss triggers immature neuronal phenotypes that are related to reduced soma size and synaptic connectivity.

## DISCUSSION AND CONCLUSIONS

We developed RTTe1 patient iPSCs to discover the effect of *MECP2e1* mutation on human neurons. RTTe1-hiPSCs retained an Xi allowing the generation of mutant *MECP2e1* neurons upon differentiation. This feature of X-inactivation during reprogramming with a preferential retention of one specific Xi in human iPS cell lines has been previously observed by us and others (Cheung et al., 2011; Pomp et al., 2011). Single cell Fluidigm arrays demonstrated that the majority of neurons were cortical in nature with an equal mixture of glutamatergic and GABAergic neurons, and the relative proportion of cell types was unaffected by *MECP2e1* or *MECP2* null mutations.

We next investigated the effect of MECP2e1 mutation on neuronal form. RTTe1-neurons displayed a soma size defect and reduced dendritic complexity in comparison to WT-neurons. The soma size was rescued with a *MECP2e1* transgene. The rescue effect was cell autonomous as only RTTe1-neurons that received the vector and not their uninfected neighbours exhibited a soma size increase. These results are consistent with the finding that nuclear size is cell autonomously regulated by Mecp2 in mouse ES cell derived neurons (Yazdani et al., 2012). The heterogeneous expression of the MECP2e1 transgene in our system allowed us to determine that transduced single cells expressing low levels of MECP2e1 had a soma size rescue comparable to WT neurons. Thus MECP2e1 rescue is level-dependent, in agreement with findings that mild overexpression of MECP2 results in neurological phenotypes (Collins et al., 2004). In contrast *MECP2e2* transgenes regulated by the same promoter in single neurons with similar low MYC staining intensity did not rescue the soma size of RTTe1 neurons. Since *Mecp2e2* transgenes can rescue certain behavioural RTT phenotypes in mice (Kerr et al., 2012), we cannot exclude the possibility that a particular level of *MECP2e2* expression during neurodevelopment may rescue some function in neurons. Taking the rescue experiments together with the reproducibility of the soma size defect in neurons derived from all 4 RTTe1-hiPSC lines, we conclude that MECP2e1 mutation reduces soma size in human neurons.

Finally, we evaluated the effect of MECP2e1 on neuronal function by identifying electrophysiological defects in RTTe1-neurons. Our finding of alterations in action potential characteristics, Na^+^ channel function and synaptic responses in RTTe1-neurons resemble those in RTT-miPSC-derived neurons (Farra et al., 2012) and extend the results from other *in vivo* RTT mouse model systems (Chao et al., 2007; Dani et al., 2005; Nelson et al., 2006; Zhang et al., 2010). Similarly, defects in synaptic function have been previously reported in human ES cell (Li et al., 2013) and hiPSC (Marchetto et al., 2010) models of RTT with loss of both MECP2 isoforms. Reduced expression level of sodium channels has been observed in several RTT models (Kim et al., 2011; Zhang et al., 2010) which may contribute to the functional defects we observed. Importantly, our finding that neuronal capacitance, an electrophysiological consequence of smaller soma size (Golowasch et al., 2009; Limon et al., 2005; Taneja et al., 2009), is reduced in RTTe1-neurons suggests that manipulation of pathways that regulate cell size may correct some of the functional defects. For instance, manipulation of the AKT/mTOR pathway was shown to increase soma size and neurite complexity of *MECP2* null human ES cell-derived neurons (Li et al., 2013) although effects on electrophysiological phenotypes were not assessed. In conclusion, our RTTe1 patient iPS cell model demonstrates that *MECP2e1* isoform mutation affects the form and function of human neurons, and shows that the cellular consequences of disease-causing alternatively spliced transcripts can be defined using patient iPS cells.

## Supplementary Material

supplement

## HIGHLIGHTS

1. MECP2e1-deficient human Rett syndrome iPS cells retain an inactive X chromosome
2. MECP2e1 controls cell-autonomous soma size and neuronal capacitance
3. MECP2e1 mutations affect neuronal Na^+^ channel and action potential properties
4. MECP2e1 mutations cause dysfunction in the frequency and amplitude of mEPSC.
