# Supplementary material for: MECP2e1 isoform mutation affects the form and function of neurons derived from Rett syndrome patient iPS cells
Source: Neurobiol Dis. Author manuscript; Available in PMC 2016 Apr 1. (PMC4380613; doi:10.1016/j.nbd.2015.01.001)
Supplement: supplement [file NIHMS662494-supplement.docx]

**Supplemental Material**

***Extended Experimental Procedures***

**Neuronal differentiation**

HiPSCs were differentiated into NPCs and neurons using the Brennand protocol and two variations (**Table S2**). Analysis of RTTe1-neurons generated with a specific protocol was compared to WT-neurons generated with the same protocol. The Brennand protocol was performed as previously described with slight modifications (Brennand et al., 2011). In brief, RTTe1-hiPSCs were detached and resuspended in N2 medium supplemented with 2 μM Dorsomorphin (Sigma) and 10 μM SB431542 (Stemgent) with medium changes everyday for one week to form CAs. N2 medium: DMEM/F-12 containing N2 (100X), MEM Non-Essential Amino Acids (NEAA) (100X), Penicillin and Streptomycin (100X) (all from Invitrogen), 2 µg ml^-1^ Heparin (Sigma), and 10 ng ml^-1^ FGF2 (R&D). Suspended CAs were then adhered on to a plastic surface coated with 0.1 mg ml^-1^ Poly-L-Ornithine (Sigma) and 20 µg ml^-1^ Laminin (Roche) (PORN/Laminin) for one week in N2 medium supplemented with 1 μg ml^-1^ Laminin to generate neuroepithelial cells (primary neural rosettes) with medium changes every other day. Primary neural rosettes were manually detached and adhered on to a plastic surface coated with PORN/Laminin for one week in NPC medium to generate a second round of neuroepithelial cells (secondary neural rosettes) with medium changes every other day. NPC medium: DMEM/F-12 containing N2 (100X), MEM NEAA (100X), Penicillin and Streptomycin (100X), B27 without Vitamin A (50X) (all from Invitrogen), 2 µg ml^-1^ Heparin (Sigma), 1 μg ml^-1^ Laminin (Roche), and 10 ng ml^-1^ FGF2 (R&D). Secondary neural rosettes were manually harvested and enzymatically dissociated using Accutase (Innovative Cell Technologies) into single cells and seeded on to a plastic surface coated with PORN/Laminin to establish NPC lines. NPCs were maintained in NPC medium with medium changes every other day and passed using Accutase with a seeding density of 1 x 10^6^ cells per well of six well-plate (Nunc) coated with PORN/Laminin. For neuronal differentiation, NPCs were adhered with a seeding density of 5 x 10^4^ per well of 24 well-plate (Nunc) with PORN/Laminin-coated coverslips in neuronal differentiation medium with medium changes every other day. Neuronal differentiation medium: Neurobasal containing N2 (100X), MEM NEAA (100X), B27 without Vitamin A (50X), Penicillin and Streptomycin (100X) (all from Invitrogen), brain-derived neurotrophic factor (BDNF), glial cell line-derived neurotrophic factor (GDNF), insulin-like growth factor-1 (IGF-1) (all from Peprotech at 10 ng ml^-1^), 1 µM N6,2’-O-dibutyryladenosine 3’,5’-cyclic monophosphate sodium salt (cAMP), 200 ng ml^-1^ Ascorbic Acid (all from Sigma), and 1 µg ml^-1^ Laminin (Roche).

The Kim protocol was performed as previously described with slight modifications (Kim et al., 2012). In brief, RTTe1-hiPSCs were detached and resuspended for 3 days in suspension with 5 mM Dorsomorphin and 10 mM SB431542 in CA medium to generate CAs. CA medium: DMEM/F12 containing 20% knockout-serum replacement, MEM NEAA, 0.1 mM beta-mercaptoethanol, Penicillin and Streptomycin (100X) (all from Invitrogen). CAs were cultured on Matrigel-coated plates (BD Biosciences) for an additional 5 days in N2 medium to form neural rosettes. Neural rosettes were isolated using pulled glass pipettes, gently dissociated mechanically, and seeded on Matrigel-coated plates in NPC medium to generate NPC lines. NPC lines were maintained similar to the B-protocol. Neuronal differentiation of NPCs was performed identical as described in the B-protocol except the neuronal differentiation medium is supplemented with 2.5 μm DAPT **(N-[N-(3,5-Difluorophenacetyl)-L-alanyl]-S-phenylglycine t-butyl ester [Sigma]).** The Brennand + DAPT protocol is performed identical to the Brennand protocol except that the neuronal differentiation medium is supplemented with **2.5 µM** DAPT.

**Neuronal Morphometric Analyses**

RTT and control NPCs were differentiated for 6 weeks *in vitro* on PORN/Laminin coated coverslips. One day before fixation, neuronal cultures were transfected with Lipofectamine 2000 (Invitrogen) so that a single neuron could be visualized amongst adjacent neurons. Neuronal cultures were given 24 hours for GFP expression before fixation. Following permeabilization, neuronal cultures were blocked for 1 hour at room temperature with 5% normal goat serum (NGS) in PBS. Neuronal cultures were incubated with rabbit anti-GFP (Invitrogen) and mouse anti-MAP2 (Sigma) primary antibodies diluted in blocking solution (see Table S4) overnight, washed 3x15 min PBS, and incubated with goat anti-rabbit- Alexa 488 (Sigma) and goat anti-mouse-Alexa 555 (Sigma) secondary antibodies diluted in blocking solution for 1 hour at room temperature. Neuronal cultures were washed 3x15 min PBS and coverslips mounted onto glass slides for imaging on a Leica DMI4000B microscope equipped with DFC340 FX camera. All images were acquired using a 20X objective lens and captured with Leica Application Suite V3.6 software. Cells positive for GFP and MAP2 were imaged and processed for dendrite morphology. Neurons were manually reconstructed with NeuronJ software. Only dendrites were included in reconstructions and axons excluded by their long morphology, which often traversed long distances beyond the field of view. Total dendrite length and number of intersections per 10μm radius determined by Sholl analysis was performed through NeuronJ and ImageJ, respectively. The number of terminal dendrite tips (projections ≥10μm in length) and the number of primary and higher order dendrites was manually counted. Student’s t-test (two-tailed) statistic was performed for total dendrite length and dendrite tip number quantifications. Multiple t-test statistic with a false discovery rate set to 5% was performed for number of dendrites per order and number of intersections by Sholl analysis. Neuronal morphology experiments were performed in triplicate and in a blinded manner.

**Electrophysiology**

Conventional whole-cell patch-clamp recordings were performed on human iPS cell-derived neurons after 6- (for recordings on action potentials) or 8- (for recordings on mEPSCs) week culture and all recordings were made using an Axopatch 1-D amplifier (Molecular Devices) at room temperature. The electrical signals were digitized at 10 kHz with a DigiData 1200 (Molecular Devices), and filtered at 2 kHz. Recording electrodes (5-8 MΩ), using micropipettes (World Precision Instruments, Inc., USA), were prepared using a P-87 pipette puller (Sutter Instrument Co., USA), and were filled, for the recordings on neuronal membrane properties and spontaneous synaptic activity, with intracellular solutions composed of (in mM, pH 7.2): 144 K^+^-gluconate, 10 KCl, 10 HEPES, 2 EGTA, and 2 Mg-ATP. The external recording solutions contained (in mM, pH 7.35): 140 NaCl, 5.4 KCl, 2 CaCl_2_, 1 MgCl_2_, 15 HEPES, and 10 glucose. Action potentials, in whole-cell current-clamp with membrane potentials of around -60 mV, were evoked by injecting a series of current steps from -5 pA to +50 pA (in 5 pA increments) for 1 s, and action potential parameters were obtained by analyzing the first evoked action potential. Voltage-gated K^+^ currents were measured at the end of 400 ms step. Current density was generated by dividing the current amplitude by cell membrane capacitance (pF), which was determined by a -10 mV voltage step and was calculated using the following equation:

Q1 + (τ x ΔI)

Capacitance =

-ΔV

Q1 (pA*ms) is the integrating area of the capacitive transient. τ (ms) is the decay time of the capacitive transient. ΔI (pA) is the plateau current by the voltage step. ΔV is pulse rate.

mEPSCs were recorded at the membrane potentials of -60 mV in whole-cell voltage-clamp. The external solutions contained (in mM, pH 7.35): 140 NaCl, 5.4 KCl, 1.3 CaCl_2_, 15 HEPES, 25 glucose, 0.0005 tetrodotoxin, 0.001 glycine; 0.01 bicuculline, and 0.01 strychnine. The intracellular solutions were composed of (in mM, pH 7.2,): 137 CsF, 15 CsCl, 10 HEPES, 10 BAPTA, 4 Mg-ATP. Mini Analysis Program (Synaptosoft Inc, NJ, USA) was used for the detection and analysis of mEPSC events.

Results were shown as mean ± SEM. [Student's *t*-test](http://en.wikipedia.org/wiki/Mann-Whitney_U_test) (two-tailed) was used for statistical analysis unless stated otherwise, and the difference was regarded to be significant when *P* value is less than 0.05.

**SUPPLEMENTAL REFERENCES**

Brennand, K. J., et al., 2011. Modelling schizophrenia using human induced pluripotent stem cells. Nature. 473**,** 221-5.

Kim, D. S., et al., 2012. Highly pure and expandable PSA-NCAM-positive neural precursors from human ESC and iPSC-derived neural rosettes. PloS one. 7**,** e39715.

**SUPPLEMENTAL FIGURE LEGENDS**

**Figure S1. RTTe1 hiPSCs karyotype analysis**.

(**A**) G-banding analysis demonstrate normal karyotypes of RTT-hiPSCs.

**Figure S2.** Single cell Fluidigm array on independently derived Δ3-4 WT and null neurons.

(**A**) Bar graph depicts the percentage of cells expressing indicated genes. Data are expressed as mean ± SEM. * p <0.05. (**B**) Analysis of cortical layer and neurotransmitter subtype from single cell Fluidigm array. Data are expressed as mean ± SEM. * p <0.05.

**Figure S3**. RTTe1 neurons have reduced dendritic complexity compared to WT controls.

(**A**) Dendrite morphology of individual WT and RTTe1 neurons visualized by transfection with EF1α-EGFP and verified for neuronal identity by MAP2 staining (red). Scale bar, 50 μm. (**B**) RTTe1 neurons have shorter total dendrite length compared to WT controls. Data are expressed as mean ± SEM. * p < 0.05. (**C**) RTTe1 neurons have reduced number of terminal dendrite tips compared to WT controls. Data are expressed as mean ± SEM. * p < 0.05. (**D**) RTTe1 neurons have unchanged numbers of primary dendrites (order 1) but reduced numbers of higher order dendrites (≥ order 2). Data are expressed as mean ± SEM. * FDR, 5%. (**E**) Sholl analysis shows that RTTe1 neurons have reduced number of intersections compared to WT controls. Data are expressed as mean ± SEM. * FDR, 5%. WT controls used in all panels are H9 and hiPSC lines derived from two unrelated healthy individuals (pluripotency characterization summarized in Table S2).

**Figure S4.** Transduction of RTTe1-NPCs and –neurons with *MECP2e1* or *MECP2e2* lentiviral vectors.

Immunocytochemistry of MECP2 and MYC in MeP-*MECP2-MYC* transduced RTTe1-NPCs (**A**) and upon differentiation into RTT-neurons (**B**). Scale bars, 44 μm.

**Figure S5.** Pluripotency characterization of SK0019_002 #7 hiPSC cell line (additional MECP2 WT control cell line).

(**A**) qRT-PCR analyses of pMXs reprogramming retroviral tansgenes (**B**) qRT-PCR of endogenous pluripotency genes in RTTe1-hiPSC lines. Data are expressed as mean ± SEM. **(C)** SK0019_002 #7 hiPSCs differentiate into three germ layers *in vitro*. Scale bars, 50 µm (immunocytochemistry). (**D**) G-banding analysis demonstrate normal karyotypes of SK0019_002 #7-hiPSCs.

**Figure S6. RTTe1-neurons exhibit electrophysiological defects.**

(**A**) Histogram shows average resting membrane potential in WT-and RTTe1-neurons. (**B**) Bar graph showing average density of voltage-gated Na^+^ currents elicited by a voltage step from −70 (the holding potential) to -20 mV in WT-neurons compared with RTTe1-neurons. (**C**) Representative traces showing spontaneous action potentials in WT- (Left) and RTTe1- (Right) neurons. (**D**) Representative traces show that action potentials were evoked by current injection (+40 pA) in a WT-neuron **(**left**)** and that TTX (0.5 μM) blocked the action potentials **(**right**)**. (**E**) Histogram showing average threshold of action potentials in WT- and RTTe1-neurons. (**F**) Bar graph shows average amplitude of action potentials for total cell lines of WT- and RTTe1 neurons. (**G**) Bar graph showing average rise time of action potentials in WT- and RTTe1 neurons. (**H**) Bar graph displaying action potential duration in WT- and RTTe1 neurons. (**I**) Histogram shows average decay time of action potentials in WT-neurons compared with RTTe1-neurons. **P*<0.05, ***P*<0.01, ****P*<0.001.

**Figure S7. WT- and RTTe1-neurons display spontaneous synaptic network activities.**

(**A**) Representative traces showing spontaneous synaptic activities in a WT-neuron.

(**B**) Representative traces showing spontaneous synaptic activities in a RTTe1-neuron.


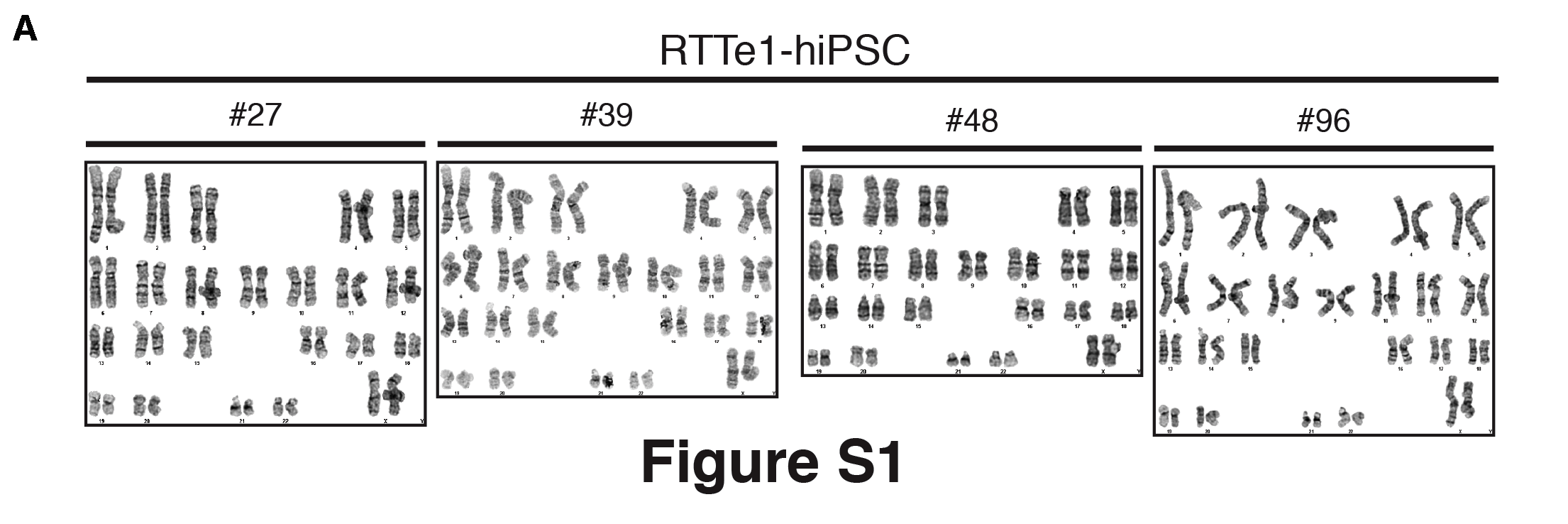


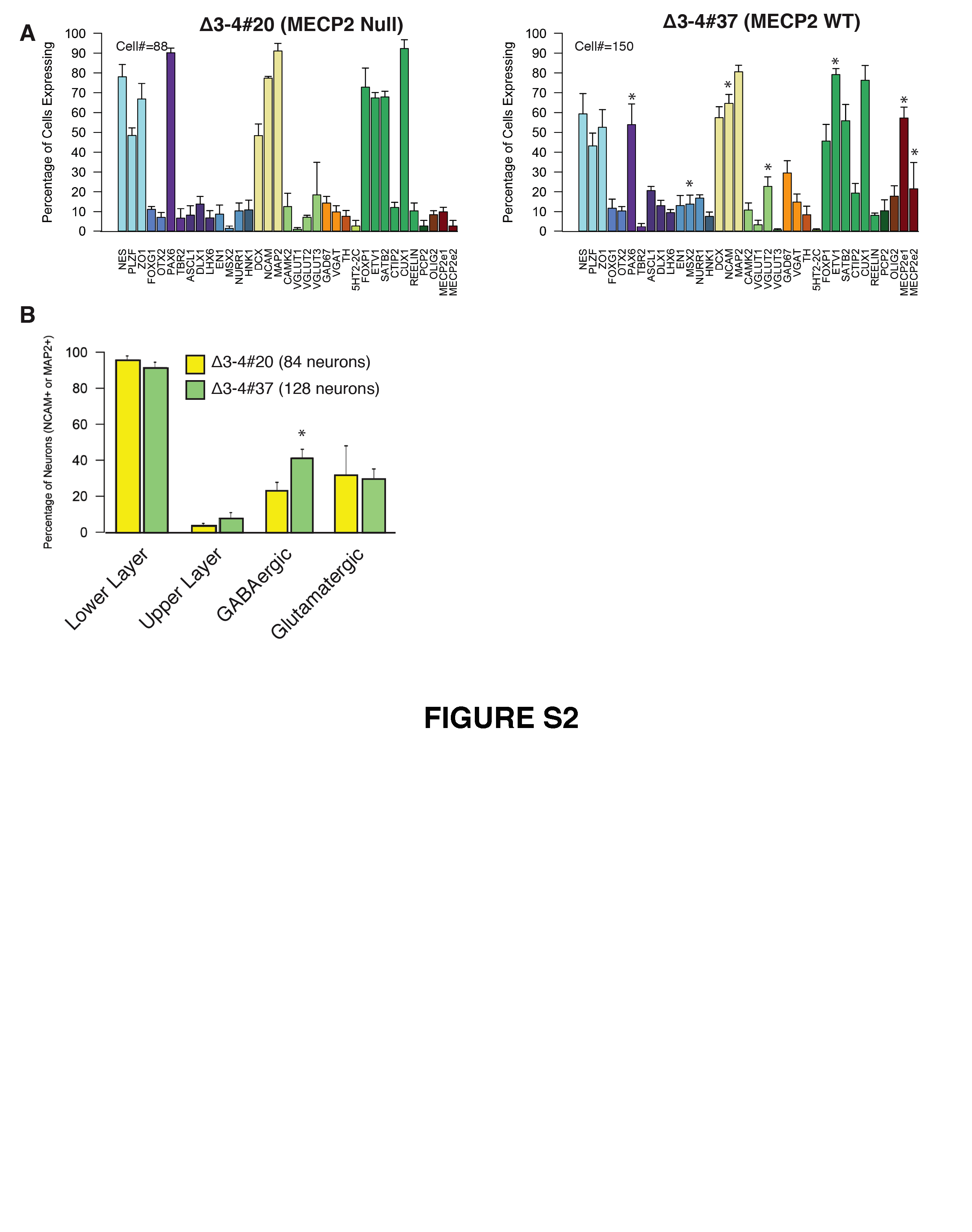

**FIGURE S3**

**Table S1.** Quantification of AR assay in RTTe1-hiPSCs and neurons.

| ± *Hpa*II & *Hha*I | Sample | Peak Area | | Corrected Peak Area | | XCI Ratio | |
| --- | --- | --- | --- | --- | --- | --- | --- |
|  |  | Allele 1 | Allele 2 | Allele 1 | Allele 2 | Allele 1 | Allele 2 |
| Undigested | RTTe1-Fibroblasts | 1392 | 1414 |  |  |  |  |
| Undigested | RTTe1-hiPSC #1 | 294 | 859 |  |  |  |  |
| Undigested | RTTe1-hiPSC #7 | 451 | 295 |  |  |  |  |
| Undigested | RTTe1-hiPSC #11 | 257 | 381 |  |  |  |  |
| Undigested | RTTe1-hiPSC #16 | 237 | 603 |  |  |  |  |
| Undigested | RTTe1-hiPSC #22 | 585 | 870 |  |  |  |  |
| Undigested | RTTe1-hiPSC #24 | 243 | 414 |  |  |  |  |
| Undigested | RTTe1-hiPSC #27 | 235 | 469 |  |  |  |  |
| Undigested | RTTe1-hiPSC #29 | 188 | 390 |  |  |  |  |
| Undigested | RTTe1-hiPSC #39 | 632 | 1277 |  |  |  |  |
| Undigested | RTTe1-hiPSC #46 | 901 | 1627 |  |  |  |  |
| Undigested | RTTe1-hiPSC #48 | 1088 | 1457 |  |  |  |  |
| Undigested | RTTe1-hiPSC #55 | 713 | 1815 |  |  |  |  |
| Undigested | RTTe1-hiPSC #57 | 1299 | 692 |  |  |  |  |
| Undigested | RTTe1-hiPSC #62 | 276 | 516 |  |  |  |  |
| Undigested | RTTe1-hiPSC #65 | 441 | 717 |  |  |  |  |
| Undigested | RTTe1-hiPSC #68 | 774 | 1581 |  |  |  |  |
| Undigested | RTTe1-hiPSC #75 | 245 | 594 |  |  |  |  |
| Undigested | RTTe1-hiPSC #79 | 418 | 1362 |  |  |  |  |
| Undigested | RTTe1-hiPSC #90 | 541 | 1114 |  |  |  |  |
| Undigested | RTTe1-hiPSC #95 | 1200 | 1748 |  |  |  |  |
| Undigested | RTTe1-hiPSC #96 | 337 | 679 |  |  |  |  |
| Undigested | RTTe1-hiPSC #108 | 896 | 1815 |  |  |  |  |
| Undigested | RTTe1-hiPSC #109 | 1479 | 659 |  |  |  |  |
| Undigested | RTTe1-hiPSC #115 | 1319 | 2100 |  |  |  |  |
| Undigested | RTTe1-neurons #27 | 251 | 356 |  |  |  |  |
| Undigested | RTTe1-neurons #39 | 815 | 1071 |  |  |  |  |
| Undigested | RTTe1-neurons #48 | 1023 | 1368 |  |  |  |  |
| Undigested | RTTe1-neurons #96 | 149 | 315 |  |  |  |  |
| Digested | RTTe1-Fibroblasts | 4240 | 1756 | 4307 |  | 71 | 29 |
| Digested | RTTe1-hiPSC #1 | 827 | 299 | 2416 |  | 89 | 11 |
| Digested | RTTe1-hiPSC #7 | 177 | 560 |  | 856 | 17 | 83 |
| Digested | RTTe1-hiPSC #11 | 622 | 211 | 922 |  | 81 | 19 |
| Digested | RTTe1-hiPSC #16 | 1160 | 205 | 2951 |  | 94 | 6 |
| Digested | RTTe1-hiPSC #22 | 910 | 194 | 1353 |  | 87 | 13 |
| Digested | RTTe1-hiPSC #24 | 1962 | 269 | 3337 |  | 93 | 7 |
| Digested | RTTe1-hiPSC #27 | 2135 | 110 | 4261 |  | 97 | 3 |
| Digested | RTTe1-hiPSC #29 | 172 | 0 | 357 |  | 100 | 0 |
| Digested | RTTe1-hiPSC #39 | 1254 | 178 | 2534 |  | 93 | 7 |
| Digested | RTTe1-hiPSC #46 | 2378 | 307 | 4293 |  | 93 | 7 |
| Digested | RTTe1-hiPSC #48 | 1107 | 242 | 1482 |  | 86 | 14 |
| Digested | RTTe1-hiPSC #55 | 1863 | 118 | 4745 |  | 98 | 2 |
| Digested | RTTe1-hiPSC #57 | 1299 | 692 | 2788 |  | 80 | 20 |
| Digested | RTTe1-hiPSC #62 | 326 | 41 | 609 |  | 94 | 6 |
| Digested | RTTe1-hiPSC #65 | 560 | 130 | 910 |  | 88 | 12 |
| Digested | RTTe1-hiPSC #68 | 1406 | 158 | 2872 |  | 95 | 5 |
| Digested | RTTe1-hiPSC #75 | 662 | 0 | 1604 |  | 100 | 0 |
| Digested | RTTe1-hiPSC #79 | 2499 | 66 | 8139 |  | 99 | 1 |
| Digested | RTTe1-hiPSC #90 | 3165 | 352 | 6510 |  | 95 | 5 |
| Digested | RTTe1-hiPSC #95 | 1169 | 294 | 1703 |  | 85 | 15 |
| Digested | RTTe1-hiPSC #96 | 1053 | 181 | 2122 |  | 92 | 8 |
| Digested | RTTe1-hiPSC #108 | 2929 | 753 | 5933 |  | 89 | 11 |
| Digested | RTTe1-hiPSC #109 | 128 | 2521 |  | 5657 | 2 | 98 |
| Digested | RTTe1-hiPSC #115 | 2549 | 298 | 4057 |  | 93 | 7­ |
| Digested | RTTe1-neurons #27 | 198 | 0 | 281 |  | 100 | 0 |
| Digested | RTTe1-neurons #39 | 2503 | 188 | 3289 |  | 95 | 5 |
| Digested | RTTe1-neurons #48 | 1523 | 54 | 2036 |  | 97 | 3 |
| Digested | RTTe1-neurons #96 | 865 | 0 | 1829 |  | 100 | 0 |

**Table S2.** Identity of RTTe1 and other hiPSC lines used in the experiments of the study.

|  | | | | RTTe1-hiPSC#27 | RTTe1-hiPSC#39 | RTTe1-hiPSC#48 | RTTe1-hiPSC#96 | Δ3-4-hiPSC #20 | Δ3-4-hiPSC#37 | SK-0019  WT |
| --- | --- | --- | --- | --- | --- | --- | --- | --- | --- | --- |
| *MECP2* cDNA sequencing | | | hiPSCs | Mutant | Mutant | Mutant | Mutant | Null pr | WT pr |  |
|  |  |  | Neurons | Mutant | Mutant | Mutant | Mutant | Null pr | WT pr |  |
| Pluripotency gene expression | | | | ✓ | ✓ | ✓ | ✓ | pr | pr | ✓ |
| Bisulfite sequencing | | | | ✓ | ✓ | ✓ | ✓ |  |  |  |
| Functional  Pluripotency | | *In vitro* | | ✓ | ✓ | ✓ | ✓ | pr | pr | ✓ |
|  |  | *In vivo teratoma* | |  | ✓ | ✓ | ✓ | pr | pr |  |
| Karyotype | | | | ✓ | ✓ | ✓ | ✓ | pr | pr | ✓ |
| Neuronal Differentiation | | | | Brennand | Kim | Brennand + DAPT | Kim | Brennand | All | Brennand |
| Fluidigm | Neurons | | | ✓ | ✓ | ✓ | ✓ | ✓ | ✓ |  |
|  | Rescued-neurons | | | ✓ |  |  |  |  |  |  |
| AR assay-iPS | | | | ✓ | ✓ | ✓ | ✓ | Pr | Pr |  |
| AR assay-neurons | | | | ✓ | ✓ | ✓ | ✓ | Pr | Pr |  |
| Soma Size | Neurons | | | ✓ | ✓ | ✓ | ✓ | Pr | ✓ |  |
|  | Rescued-neurons | | | ✓ |  |  |  |  |  |  |
| Electrophysiology | | | | ✓ | ✓ | ✓ | ✓ | ✓ | ✓ | ✓ |
| Neuronal Morphometry | | | | ✓ | ✓ |  | ✓ | H9 | SK144 | SK0186 |

| **Primer** | **Forward (5’ 🡪 3’)** | **Reverse (5’ 🡪 3’)** |
| --- | --- | --- |
| **Primers used for Fluidigm, cDNA sequencing and qRT-PCRs:** | | |
| *MECP2-V1* | GTAAAAGCCGTCCGGAAAAT | GCTTAAGCTTCCGTGTCCAG |
| *NES* | GGCGCACCTCAAGATGTCC | CTTGGGGTCCTGAAAGCTG |
| *PLZF* | GGGACTTTGTGCGATGTGGT | ATTGCGGTGGAAGAGGATCTC |
| *ZO1* | AGTCCCTTACCTTTCGCCTGA | TCTCTTAGCATTATGTGAGCTGC |
| *FOXG1* | GCCACAATCTGTCCCTCAACA | CGGGTCCAGCATCCAGTAG |
| *OTX2* | CAACCGCCTTACGCAGTCAA | GGGGTGCAGCAAGTCCATAC |
| *ZIC1* | GTTCGGAGCACTATGCTGC | TTGCACGACTTTTTGGGGTTG |
| *PAX6* | ATGTGTGAGTAAAATTCTGGGCA | GCTTACAACTTCTGGAGTCGCTA |
| *TBR2* | CCGGGCACCTATCAGTACAG | GGTTGCACAGGTAGACGTG |
| *ASCL1* | TCTTCGCCCGAACTGATGC | CAAAGCCCAGGTTGACCAACT |
| *DLX1* | CCATGCCAGAAAGTCTCAACA | GGCCCAAACTCCATAAACACC |
| *NKX2.1* | AGCACACGACTCCGTTCTC | GCCCACTTTCTTGTAGCTTTCC |
| *LHX6* | TGAGAGTCAGGTACAGTGCG | GCCCATCCATATCGGCTTTGA |
| *EN1* | GAGCGCAGGGCACCAAATA | AATAACGTGTGCAGTACACCC |
| *MSX2* | CACCCTGAGGAAACACAAGAC | AACTCTGCACGCTCTGCAAT |
| *NURR1* | TGTGTTCAGGCGCAGTATGG | TCCCGAAGAGTGGTAACTGTAG |
| *HNK1* | CCTGGCGTGGTCTACTTCG | GCAGGTTGACGGCAAATCC |
| *DCX* | CCTTGGCTAGCAGCAACAGT | CCACTGCGGATGATGGTAA |
| *NCAM* | ACATCACCTGCTACTTCCTGA | CTTGGACTCATCTTTCGAGAAGG |
| *MAP2* | CTGCTTTACAGGGTAGCACAA | TTGAGTATGGCAAACGGTCTG |
| *CAMK2* | AAACTGAAGGGAGCCATTCTCA | GAGGATTCCATTAACTGAACGCT |
| *VGLUT1* | CGACGACAGCCTTTTGTGGT | GCCGTAGACGTAGAAAACAGAG |
| *VGLUT2* | GGGAGACAATCGAGCTGACG | CAGCGGATACCGAAGGAGATG |
| *VGLUT3* | AAACCGGAAATTCAGACAGCA | CCAAAGACCCTGTTAGCAGCA |
| *GAD67* | GCCAGACAAGCAGTATGATGT | CCAGTTCCAGGCATTTGTTGAT |
| *VGAT* | CCGAGTGGTGAACGTAGCG | GTGGCGATAATGGACCAGGAC |
| *TH* | GCCCTACCAAGACCAGACGTA | CGTGAGGCATAGCTCCTGA |
| *5HT2-2C* | TCTTAATGTCCCTAGCCATTGCT | TACCGATCCAGCGATATAGCG |
| *FOXP1* | AGACAAAAAGTAACGGTTCAGCC | CGCACTCTAGTAAGTGGTTGC |
| *ETV1* | CTGGATGACCCGGCAAATTCT | CCTCTTCAGGCTCAATCAGTTT |
| *SATB2* | TCTCCCCCTCAGTTATGTGAC | AGGCAAGTCTTCCAACTTTGAA |
| *CTIP2* | TGGGTGCCTGCTATGACAAG | GGCTCGGACACTTTCCTGAG |
| *CUX1* | GCTCTCATCGGCCAATCACT | TCTATGGCCTGCTCCACGT |
| *REELIN* | TCCGGGACAAGAATACCATGT | CCAAATCCGAAAGCACTGGAA |
| *PCP2* | AGAGGCCAGCAGAAAAGTGACT | GTGGCTCAGCAGATTGAAGAA |
| *OLIG2* | GGACAAGCTAGGAGGCAGTG | ATGGCGATGTTGAGGTCGTG |
| *MECP2e1* | AGGAGAGACTGGAAGAAAAGTC | CTTGAGGGGTTTGTCCTTGA |
| *MECP2e2* | CTCACCAGTTCCTGCTTTGATGT | CTTGAGGGGTTTGTCCTTGA |
| *MECP2* | ACACATCCCTGGACCCTAATGA | TGGGCTTCTTAGGTGGTTTCTG |
| *18S* | GATGGGCGGCGGAAAATAG | GCGTGGATTCTGCATAATGGT |
| *GAPDH* | CATGAGAAGTATGACAACAGCCT | AGTCCTTCCACGATACCAAAGT |
| **Primers used for bisulfite sequencing:** | | |
| *pMXs-LTR* | TTTATTTGTAGGTTTGGTAAGTTAGTTTAA | ATAATCCCTAAACAAAAATCTCCC |

**Table S3.** Sequences of primers used in the study.

**Table S4.** List of antibodies used in the study.

| Antibody | Company | Catalogue Number | Dilution |
| --- | --- | --- | --- |
| TUJ1 | Chemicon | MAB1637 | 1:200 |
| SMA | Invitrogen | 18-01106 | 1:200 |
| GATA4 | Santa Cruz | SC-9053 | 1:200 |
| MECP2 | Sigma | M6818 | 1:1000 |
| c-MYC | Santa Cruz | Sc-789 | 1:200 |
| MAP2 | Millipore | AB5622 | 1:1000 |
| MAP2 | Sigma | MI406 | 1:3000 |
| GFP | Invitrogen | A11122 | 1:1000 |
| MECP2 (W. Blot) | Abcam | Ab2828 | 1:2000 |
| MECP2 (W. Blot) | Millipore | 07-013 | 1:1500 |
| Pan H3 | Cell signalling | 9715 | 1:5000 |
